# Morphometric and Gray-Level Texture Analyses of Postmortem Injuries Using ImageJ: An Exploratory Study

**DOI:** 10.7759/cureus.113262

**Published:** 2026-07-23

**Authors:** Rohit K Singh, Shiuli Rathore, Rakhi Rajput, Garima Sehgal, Shyamchand Chaudhary, Raghvendra Singh, Ashutosh D Tiwari, Sachin K Tripathi, Anoop K Verma

**Affiliations:** 1 Forensic Medicine and Toxicology, King George’s Medical University, Lucknow, IND; 2 Forensic Medicine and Toxicology, King George's Medical University, Lucknow, IND; 3 Anatomy, King George's Medical University, Lucknow, IND; 4 Medicine, King George's Medical University, Lucknow, IND; 5 Forensic Medicine and Toxicology, Era's Lucknow Medical College and Hospital, Lucknow, IND; 6 Photography, Central Forensic Science Laboratory, Delhi, IND; 7 Forensic Medicine, King George’s Medical University, Lucknow, IND

**Keywords:** exploratory study, forensic image analysis, gray-level analysis, imagej, morphometry, postmortem injuries

## Abstract

Background

The quantitative methods of image analysis can offer objective data on the structure of the injury, its edge architecture, geometrical properties (such as length, width, area, perimeter, and solidity), and gray-level textures.

Aim

To explore the utility of quantitative morphometric and gray-level texture analyses of postmortem injuries.

Materials and methods

This pilot study was performed in a cross-sectional manner and used an exploratory design in the Department of Forensic Medicine & Toxicology, King George’s Medical University (KGMU), Lucknow. A total of 383 injury images in medico-legal autopsies were studied with the help of the ImageJ software (version 1.53, National Institutes of Health, Bethesda, MD, USA). Parameters such as length, width, area, perimeter, solidity, and gray-level texture were evaluated. The statistical analysis comprised descriptive statistics, comparative analysis, correlation analysis, multicollinearity, exploratory logistic regression, and receiver operating characteristic (ROC) analysis.

Results

Notable differences were seen for the area, perimeter, solidity, and gray-level standard deviation (SD) between injury groups (p<0.05). There was a notable positive correlation between the area and perimeter (r=0.88). Regression analysis showed statistical significance for the area, SD, and solidity. The exploratory analysis yielded good discrimination power with an area under the curve (AUC) of 0.84 (95% confidence interval (CI): 0.77-0.90).

Conclusion

The above exploratory investigation shows that image-based techniques such as quantitative morphometry and grayscale texture analysis using the ImageJ software could potentially offer reliable, quantifiable, and objective data about postmortem trauma. Nonetheless, image-based measurements must only be used as a supplementary tool to the traditional forensic evaluation and cannot in any case replace the gross examination and expert opinion of a forensic pathologist. There is a need for large-scale, multicenter investigations with external validation before the said techniques can be considered for forensic use.

## Introduction

Postmortem injury assessment and interpretation can be considered a very important part of forensic science and an important element in the clarification of cause and manner of death. The quantitative methods of image analysis can offer objective data concerning the structure of the injury, its edge architecture, geometrical properties (such as length, width, area, perimeter, and solidity), and gray-level textures [1]. Forensic professionals have used visual inspection, gross pathology, and their own expertise to assess injuries such as abrasions, contusions, cuts, ligature marks, and gunshot wounds [2]. While traditional forensic examination has been the bedrock of forensics, visual assessment by nature is subjective and can differ between examiners based on experience, surroundings, and image quality [3]. This subjectivity can affect forensic documentation and interpretations, especially when there is subtlety or overlap among injury characteristics [4].

The recent developments in digital imaging and computational image analysis techniques have created new possibilities for quantitative evaluation in forensic sciences [5]. Digital image analysis provides the ability to extract quantifiable information about morphology and texture from photographs and thereby minimize reliance on subjective analysis [6]. In forensic applications, the use of quantifiable measurements through imaging can help document injuries and make scientific observations [7].

The existing software tools for image analysis include NIH ImageJ, a popular image processing software developed by the National Institutes of Health (Bethesda, MD, USA). The software is capable of quantitatively analyzing the morphological parameters such as length, width, area, perimeter, and shape of a body structure [8,9]. These features can help to understand additional aspects related to the geometrical, surface, and structural properties of an injury [10].

Morphometrics is a useful method in evaluating images from a quantitative perspective. Parameters such as area and perimeter provide information about the size and shape of injuries; meanwhile, solidity indicates the regularity and tightness of the injury margins [11]. Lesions with irregular or discontinuous margins tend to have smaller solidity compared with injuries having smooth boundaries [12]. These parameters might assist forensic evaluators in quantifying observations when discrimination proves challenging visually [13].

Moreover, along with morphometric analysis, gray-level texture analysis has become significant in evaluating images digitally [14]. Gray-level features can be extracted based on variations in intensity values of pixels in a chosen ROI and might indicate tissue heterogeneity and texture properties of the image [15]. Features such as minimum gray level, maximum gray level, median gray level, and standard deviation (SD) can demonstrate variations in tissue structure and image intensity [16]. Some previous research has indicated that variations in texture could help detect minor differences in tissue structures and injuries [17].

Forensic photographic standards are yet another vital prerequisite for a meaningful image-based study [18]. Camera perspective, lighting conditions, exposure, and scaling can play an important role in influencing any morphometric or texture measure [19]. Thus, the implementation of forensic scales such as the ABFO No. 2 scale (American Board of Forensic Odontology; Florida, USA) in combination with standardized imaging conditions has been suggested as one means of reducing measurement errors [20].

Although there has been increasing research activity in forensic image analysis, existing literature is relatively sparse and uncoordinated. Previous research has been centered around discrete measurements, specific injuries, and imaging methods used for experiments rather than a comprehensive quantification [21]. Moreover, few papers have been published by resource-limited forensic centers, wherein computational methodologies are in development [22]. Moreover, issues related to reproducibility, observer bias, multicollinearity between morphological measures, and the absence of external validation remain barriers to forensic applications [23].

In the current study, parameters such as size, shape (length, width, area, perimeter, and solidity), and grayscale properties (minimum gray level, maximum gray level, median gray level, and SD) of the injuries were measured by standardized quantitative morphometric and texture analyses on digitized images.

The current study should be seen more as an exploratory study to generate hypotheses rather than as a conclusive study to validate any diagnosis. The results obtained could form the basis of further studies that would involve standardized imaging procedures, automatic image analysis algorithms, and external validation sets.

The aim of the current study was to evaluate the potential of quantitative morphometric and gray-level texture analysis of post-mortem injuries utilizing the ImageJ software and to determine whether these parameters may serve as objective additional information in forensic documentation of injuries.

## Materials and methods

Study design and setting

This current research was undertaken as a prospective cross-sectional exploratory study in the Department of Forensic Medicine and Toxicology at King George’s Medical University (KGMU), Lucknow, Uttar Pradesh, India. In this study, the aim was to assess the morphometric as well as gray-level texture features of postmortem injuries using standardized digital image analysis techniques. This study was planned as an exploratory study to generate hypotheses rather than validate diagnoses. Standardized image acquisition and quantitative image analysis were stressed in this research.

Ethical approval

Ethics clearance was provided by the Institutional Ethics Committee of King George’s Medical University, Lucknow (IEC No.: 104th ECM II B-PhD/P5). Anonymization was done for all medico-legal images used in this study. No personal details were stored in the data bank. The images were used for educational and research purposes according to medico-legal norms set by the institution.

Study population

The study sample consisted of medico-legal autopsy cases reported to the Department of Forensic Medicine and Toxicology at King George’s Medical University, Lucknow, during the study period. The unit of observation in this study is the image of injury, not the autopsy case. A total of 383 autopsy images depicting injuries that met the set criteria were used for quantitative image analysis. Images selected for analysis depicted various forms of injury as reported in medico-legal autopsies. In cases with multiple injuries in a single autopsy, if each injury met the inclusion criteria, more than one image per case could be included. This was because each injury had to be observed independently. The present study aimed at exploring image analysis; hence, all images meeting the inclusion criteria during the study period were used.

Sampling technique and sample size

Consecutive sampling methodology was used in this study. Any image showing injuries meeting the eligibility criteria as well as having image quality criteria was included. Because the focus of the study was exploratory in terms of assessing morphometric and grayscale properties of injuries sustained after death, the injury image was taken as the basic unit of analysis. A total of 383 eligible images of injuries were used for analysis. If several images were obtained from the same autopsy case, those images that depicted different injuries were included. As a result of the exploratory nature of the research and lack of data for forensic morphometric studies using imaging, no sample size calculations were conducted.

Inclusion criteria

We included medico-legal autopsies where there were observable injuries to the body with easily identifiable margins to allow for proper measurement. Only injuries whose margins could be clearly determined were used. For inclusion in the study, good pictures of the body and an ABFO No. 2 forensic scale had to be present in the picture.

Exclusion criteria

The study left out cases where injuries were too decomposed or badly burned to be assessed properly. We also did not use photos that were fuzzy, poorly lit, or just plain distorted. Any pictures where the injuries were changed due to stuff that happened after death were not used either. In addition, we excluded anything without a scale for proper measurement. Lastly, if environmental damage messed with how the images could be interpreted, those were removed as well.

Operational definitions

Abrasion was described as a superficial injury where there is a loss of integrity or damage to the epidermis but no substantial amount of subcutaneous bleeding. Contusion was described as a closed soft-tissue injury where there was subcutaneous bleeding but no disruption to skin integrity. Because the subgroups were not adequately sized, gunshot and electrocution injuries were grouped under “other injuries."

Image acquisition protocol

To control the effect of confounding variables, the forensic photography process was strictly standardized for this study. For the study, all the photographs used had been taken using the same Nikon DSLR camera (Nikon Corporation; Tokyo, Japan) with consistent imaging conditions. In particular, the Nikon camera was always placed at a 90° angle perpendicular to the injury surface, at the same working distance where possible. Consistent camera settings were adopted (ISO 100-200, aperture f/8-f/11, white balance set to fixed, and exposure). Each photograph had the ABFO No. 2 forensic scale placed in it. The exclusion criteria included blurred photographs, out-of-focus photographs, photographs with shadows, reflections, overexposure, underexposure, misplacement of scale, and an oblique angle of the camera. To reduce variability resulting from image processing, all images went through the same process of calibration, grayscale conversion, and normalization. Besides, highly decomposed bodies, burned wounds, and wounds with vague margins were excluded from the study.

Image processing and analysis

**Figure 1:**
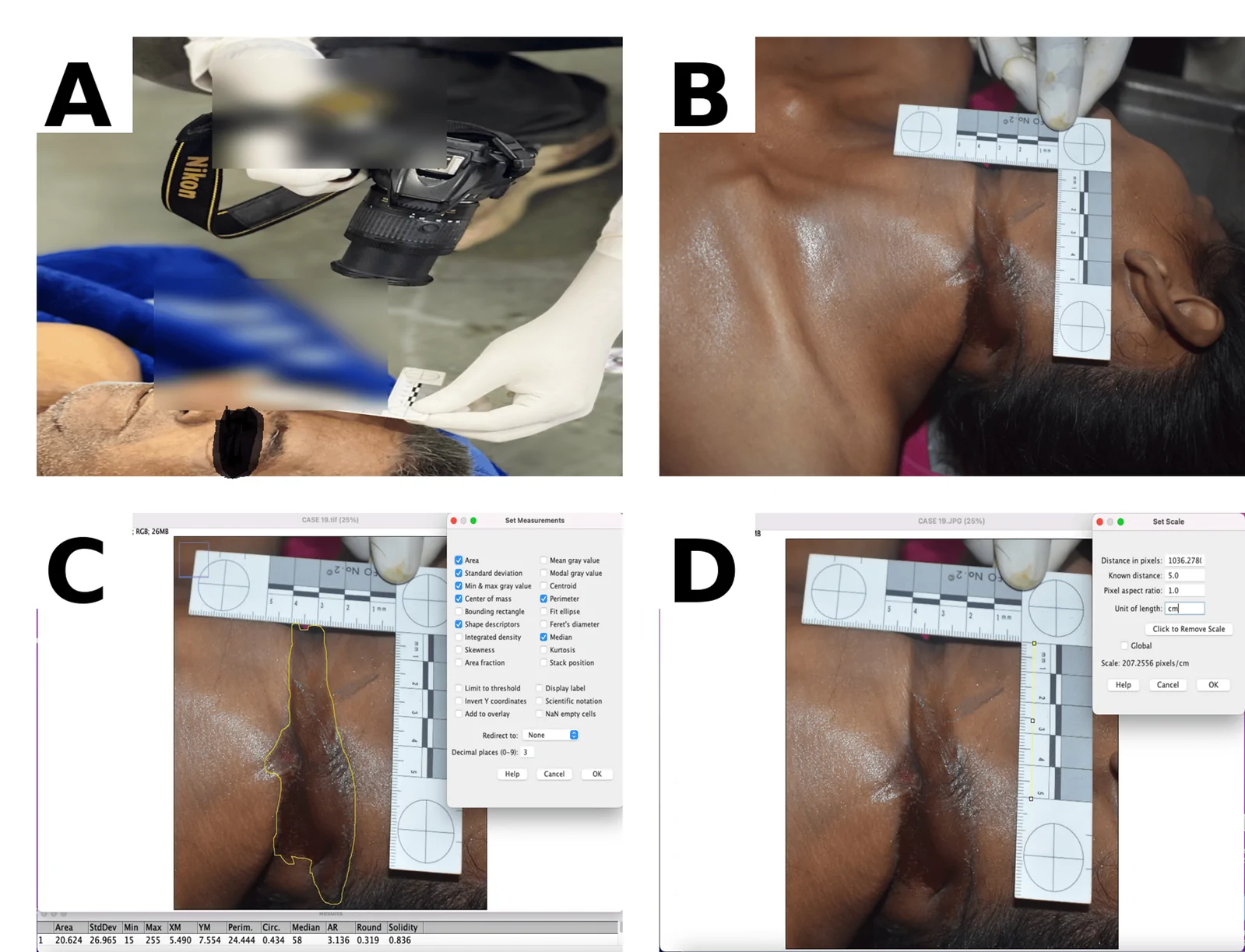
Standardized forensic photography setup showing injury documentation using a DSLR camera with ABFO No. 2 scale for calibration under controlled conditions. Standardized forensic photography and ImageJ-based image analysis workflow. (A) Documentation of postmortem injury using a DSLR camera under controlled photographic conditions. (B) Injury photograph showing placement of the ABFO No. 2 forensic scale for calibration. (C) Manual delineation of the region of interest (ROI) in ImageJ software for morphometric analysis. (D) Calibration and quantitative measurement of injury dimensions using ImageJ software following ROI selection.

Image processing in digital images was conducted using the ImageJ software (version 1.53, National Institutes of Health, Bethesda, MD, USA). All obtained images were subjected to uniform preprocessing before being quantitatively analyzed. Preprocessing consisted of conversion into grayscale, calibration by ABFO No. 2 forensic scale, and normalization of intensity values. Standardized procedures for image preprocessing were used for all images in the study. The standardized forensic photography and ImageJ-based image analysis workflow is illustrated in Figure 1.

Region of interest (ROI) selection

The selection of region of interest (ROI) was done manually based on standardized criteria set forth beforehand. The edges of the injury were precisely demarcated such that uninvolved tissue adjacent to the injury was not incorporated. In order to minimize subjectivity on the part of the observer, the selection process was done independently by two observers who had knowledge of ImageJ image analysis methods. Differences in opinion among observers were reconciled before measurements were recorded.

Observer reliability assessment

Before conducting any image measurements, both observers were equally trained on how to choose ROI, define the borders of injuries, calibrate the images, and measure using the ImageJ software. A standard operating procedure (SOP) was strictly followed during image measurements. Inter-observer reproducibility was evaluated using the intraclass correlation coefficient (ICC), which proved highly reliable for area (ICC=0.88), perimeter (ICC=0.84), and solidity (ICC=0.81).

Parameters evaluated

The study investigated both morphometric and gray-level textural measurements derived from digital image processing. The morphometric measurements consisted of length, width, area, perimeter, and solidity, all chosen for evaluating the geometry, size, and borders of injuries. On the other hand, gray-level textural measurements included minimum gray level, maximum gray level, median gray level, and SD of gray levels. These measurements were considered for assessing pixel intensity and tissue heterogeneity within the selected injury areas.

Confounding variables

A number of steps were taken to minimize the influence of possible confounding variables on the results of the study. Similar photo-taking conditions, same camera settings, similar calibration processes, and same image processing methods were applied to all cases. All cases that showed advanced decomposition, burning, environmental deterioration, non-defined injury borders, and poor images were rejected. Only those injuries that had defined borders and were adequate for morphometric analysis were used. While it was impossible to rule out all the possible effects of anatomical region, skin color, time since death, and natural heterogeneity of an injury, some steps were taken toward minimizing their influence.

Data management

All the metrics that were extracted through morphometrics and texture analysis were documented using Microsoft Excel (Redmond, Washington, USA) sheets, where the cases were assigned anonymous identification numbers to ensure confidentiality. This database was validated for any missing data, data entry errors, or inconsistencies before undertaking any statistical analysis.

Statistical analysis

Statistical analysis was conducted using IBM SPSS Statistics v. 26.0 (released 2018; IBM Corp., Armonk, NY, USA) and R software v. 4.3 (R Foundation for Statistical Computing, Vienna, Austria). Continuous data are summarized using mean ± SD, median, interquartile range (IQR), and minimum-maximum range where appropriate. Statistical differences between morphometric and grayscale texture parameters across the injury groups were analyzed using one-way analysis of variance (ANOVA) with Bonferroni post-hoc adjustment. Correlations were determined using Pearson's correlation coefficient analysis. The level of multicollinearity was assessed by the variance inflation factor (VIF). Binary logistic regression was used to find the best predictors of injuries, while the performance of the regression model was checked using the Omnibus test, Nagelkerke R², the Hosmer-Lemeshow test, and the classification accuracy of the model. Receiver operating characteristic (ROC) curve analysis was used to assess the discrimination potential of significant predictors and the logistic regression model; the area under the curve (AUC) was calculated, and sensitivity, specificity, and 95% confidence interval (CI) were determined. Inter-observer reliability was assessed by the ICC. A p-value≤0.05 was set to indicate statistical significance.

## Results

The majority of the patients included in this study sustained injuries from road traffic accidents (RTAs), representing 81.98% of the patients, whereas the other type that formed the highest frequency was hanging injuries, accounting for 15.14% of the total study population. Firearm injuries and electric shock were uncommon and, therefore, could not be taken into consideration for comparative statistical purposes (Table 1). RTA injuries constituted the majority of cases in the present study. Hanging injuries represented the second largest category, whereas firearm and electrocution injuries were comparatively infrequent. The unequal group distribution should be considered during the interpretation of comparative statistical findings, and it limits broader generalizability.

**Table 1:** Distribution of Injury Types (n=383)

Injury type	Frequency (n)	Percentage (%)
Road traffic accident (RTA)	314	81.98
Hanging	58	15.14
Firearm injury	7	1.83
Electrocution	4	1.04
Total	383	100

Descriptive statistics of morphometric and gray-level texture parameters are summarized in Table 2. Substantial variability was observed in morphometric and texture-based parameters, indicating heterogeneity in injury morphology and image characteristics. Area and perimeter demonstrated broad ranges, reflecting variability in injury extent and geometry. Variation in gray-level SD suggests differences in tissue texture and pixel intensity distribution among injury categories.

**Table 2:** Descriptive Statistics of Morphometric and Gray-Level Parameters (n=383) SD: standard deviation; IQR: interquartile range

Parameter	Mean ± SD	Median (IQR)	Minimum-maximum
Length (cm)	7.53 ± 5.11	6.40 (3.2-10.5)	0.50-25.00
Width (cm)	2.66 ± 2.21	2.10 (1.0-3.8)	0.10-10.00
Area (cm²)	18.45 ± 24.86	9.80 (2.5-24.2)	0.03-168.70
Perimeter (cm)	18.01 ± 16.12	12.40 (4.8-24.5)	0.50-86.00
Minimum gray value	41.52 ± 23.41	38 (22-57)	0-120
Maximum gray value	254.82 ± 2.91	255 (254-255)	240-255
Median gray value	96.24 ± 18.33	94 (82-108)	42-155
Standard deviation	32.48 ± 12.76	30.5 (24-39)	8-78
Solidity	0.79 ± 0.11	0.81 (0.73-0.88)	0.42-0.98

Comparative analysis of morphometric and gray-level parameters among injury groups is presented in Table 3. Statistically significant differences were found in length, width, area, perimeter, SD, and solidity. The highest F-value was for solidity (F=11.94, p<0.001), showing big differences in boundary regularity between injury groups. RTA injuries were larger in dimension than hanging and other injuries. The gray-level SD varied significantly, too, pointing to differences in tissue heterogeneity and image texture characteristics among the groups.

**Table 3:** Comparison of Morphometric and Gray-Level Parameters Between Injury Groups Using One-Way ANOVA One-way ANOVA; *statistically significant at p<0.05. SD: standard deviation; ANOVA: analysis of variance; RTA: road traffic accident

Parameter	RTA (mean ± SD)	Hanging (mean ± SD)	Other injuries (mean ± SD)	F-value	df	p-value
Length (cm)	7.82 ± 5.22	6.11 ± 4.31	5.90 ± 4.84	3.21	2,380	0.041*
Width (cm)	2.79 ± 2.18	2.08 ± 1.91	1.95 ± 1.70	3.07	2,380	0.048*
Area (cm²)	20.62 ± 25.88	10.54 ± 14.63	8.22 ± 12.81	5.96	2,380	0.003*
Perimeter (cm)	19.48 ± 16.92	11.94 ± 10.88	10.52 ± 11.10	5.17	2,380	0.006*
Median gray value	97.84 ± 18.11	90.40 ± 17.02	88.33 ± 15.20	2.49	2,380	0.084
Standard deviation	34.12 ± 12.91	27.08 ± 10.44	24.91 ± 9.62	4.56	2,380	0.011*
Solidity	0.82 ± 0.09	0.71 ± 0.11	0.68 ± 0.10	11.94	2,380	<0.001*

Assessment of multicollinearity among predictor variables using VIFs is shown in Table 4. The multicollinearity analysis showed that the predictor variables had acceptable correlations. All VIF values stayed below 5.0, so there was no severe multicollinearity. While the area and perimeter had somewhat higher VIFs, they were still within acceptable limits. This suggests the regression model is stable and good for further analyses.

**Table 4:** Multicollinearity Assessment Using Variance Inflation Factor (VIF)

Variable	VIF	Tolerance
Length	2.81	0.356
Width	2.42	0.413
Area	4.18	0.239
Perimeter	4.72	0.212
Standard deviation	1.64	0.61

Results of exploratory logistic regression analysis are presented in Table 5. Exploratory binary logistic regression found that area, gray-level SD, and solidity predict injury differentiation. Solidity, specifically, had the strongest link-Wald χ² equals 7.61, odds ratio of 8.24, 95% CI of 1.82 to 37.24, and p of 0.006. This means that injuries with smoother edges are more likely to fall into certain categories. Overall, the model worked great, with an Omnibus χ² of 21.84, 3 degrees of freedom, and p less than 0.001, showing the variables helped a lot in classifying injuries. With a Nagelkerke R² of 0.318, about 31.8% of the variation in injury types can be chalked up to these factors. Also, the Hosmer-Lemeshow test was not significant (χ² = 6.12, p = 0.634), showing a well-calibrated model, while 79.6% overall accuracy showed good prediction.

**Table 5:** Exploratory Logistic Regression Analysis and Overall Model Performance Binary logistic regression; *statistically significant at p<0.05. SE: standard error; CI: confidence interval

Variable/model statistic	β Coefficient	SE	Wald χ²	Odds ratio (OR)	95% CI	p-value
Area (cm²)	0.021	0.01	4.13	1.02	1.001-1.04	0.042*
Standard deviation	0.062	0.026	5.61	1.06	1.01-1.11	0.018*
Solidity	2.11	0.765	7.61	8.24	1.82-37.24	0.006*
Overall model statistics						
Omnibus test (χ²)	-	-	21.84	-	-	<0.001*
Degrees of freedom (df)	-	-	3	-	-	-
Nagelkerke R²	-	-	0.318	-	-	-
Hosmer-Lemeshow χ²	-	-	6.12	-	-	0.634
Classification accuracy (%)	-	-	79.6	-	-	-

Diagnostic performance of selected parameters obtained through ROC analysis is summarized in Table 6. The ROC analysis showed that the combined predictive model worked well, with an AUC of 0.84 (95% CI: 0.77-0.90). Of the individual predictors, solidity was the best (AUC=0.81), followed by gray-level SD and area. All predictors demonstrated discriminatory performance significantly better than chance, backed by the statistically significant Z statistics. So, these quantitative morphometric and texture parameters could be useful tools to support forensic injury classification.

**Table 6:** ROC Curve Analysis of Significant Predictors *Statistically significant at p<0.05. ROC: receiver operating characteristic; AUC: area under the curve

Parameter	AUC (95% CI)	Z Statistic	Sensitivity (%)	Specificity (%)	p-value
Solidity	0.81 (0.73-0.88)	7.82	79.4	76.2	<0.001*
Standard deviation	0.74 (0.66-0.82)	5.61	71.2	69.8	<0.001*
Area	0.70 (0.61-0.79)	4.12	68.4	65.7	0.001*
Combined model	0.84 (0.77-0.90)	8.93	82.1	78.4	<0.001*

## Discussion

Our exploratory study used the ImageJ software to look at postmortem injuries through quantitative morphometric and gray-level texture analysis. We noticed key differences in parameters such as area, perimeter, solidity, and gray-level texture. This shows that these objective image-based measurements could give extra information for forensic injury assessments and record-keeping.

Digital image analysis is now a key tool in cutting down on observer dependency in forensics. According to Moscalu et al. (2023) [24], computational methods can objectively pull out exact data from biological images, both shape-related and texture-related, that might boost the reliability of results compared to just eyeballing them. This study backs that up, too; the ImageJ tools did a great job at measuring injury details, which showed clear statistical differences between the types of injuries.

In this study, a key finding was that RTA injuries had larger areas and perimeters compared to other types of injuries. This aligns with the findings of Baruah et al. (2025) [25], which showed that quantitative shape features such as area and perimeter help distinguish between different forensic image types. Though they looked at ballistic evidence, the idea of using these measurable traits was pretty much the same. It reinforces how useful morphometric analysis can be in forensic settings.

There is a strong positive link between area and perimeter (r=0.88), matching up with established morphometric rules. Typically, bigger injuries have longer borders, causing those measures to go up together. This connection is backed by earlier quantitative image-analysis studies that found these geometric variables to be pretty interdependent. To check for model instability, they used VIF analysis. Fortunately, all VIF values stayed under 5, meaning the multicollinearity was okay. So, the regression model is good to go.

Maintaining standardization in forensic photography is key to getting accurate measurements. Ferrucci et al. (2016) [26] pointed out that proper calibration scales and set procedures are crucial for forensic photo analysis. For our study, we adhered to these standardized methods and used the ABFO No. 2 forensic scale for all pictures. Doing this probably reduced errors and made our quantitative assessments more reliable. Standardized procedures really matter because changes in camera angle, lighting, or scale placement could seriously affect measurements of both shape and texture.

Among all the variables looked at, solidity showed the biggest differences between injury types and was the strongest predictor in the analysis. Solidity tells us about the regularity and compactness of a shape's edge. If solidity is high, it means the injury edges are smooth and regular. Low solidity, in contrast, suggests rough or broken edges. Patra et al. (2023) [27] pointed out that understanding wound shapes and edges is crucial for forensic work. Our current findings build on this, showing that we can actually measure these features objectively through digital image analysis, instead of just relying on what we see with the naked eye.

Gray-level texture analysis was really helpful in our investigation too. It showed certain key differences among injury groups, and the gray-level SD was statistically significant in logistic regression analysis. These texture parameters show how varied the tissue is inside the region we look at. 

Harris et al. (2018) [14] found that texture-based features can effectively highlight structural differences in biological tissues. Wilk et al. (2025) [16] showed that advanced imaging can identify bruises and soft-tissue injuries well. Our results fit with this growing evidence that grayscale texture analysis could very well supplement traditional morphometric measurements for better image interpretation in forensics.

The exploratory logistic regression model found that area, gray-level SD, and solidity are key in figuring out injuries, with solidity showing the strongest connection. Solidity has the highest OR, meaning it associates strongly with injury classification. These results suggest that combining multiple image-based features may improve the differentiation of injury types. Other studies in predictive image analysis came to similar conclusions: combining shape-based and texture-based factors tends to boost classification more than using single parameters. Similarly, Moscalu et al. (2023) [24] highlighted the potential value of multiparametric approaches that integrate multiple image-derived features in predictive image modeling.

The current study also assessed inter-observer agreement and demonstrated high reliability for area, perimeter, and solidity, supporting the reproducibility of the ImageJ-based measurement approach. This consistency is crucial because reproducibility is necessary for any quantitative image-analysis technique to be used in forensics. In summary, the research shows that analyzing shapes and textures gives reliable measurements. These could back up standard ways of examining postmortem injuries. Yet, we should not view these results as solid proof on their own. Instead, they add to the case for using digital image analysis in forensic work. Furthermore, they hint at possible uses in future technology, such as automatic injury classifiers and artificial intelligence (AI) tools that assist forensic experts.

This study has some important limitations to keep in mind. It is exploratory and took place at one location, where categories of injuries were not evenly distributed; there were way more RTA cases than others. With fewer firearm and electrocution injuries, there is less statistical power for a detailed breakdown. Although manual ROI selection had good agreement between observers, it can still vary from person to person. Also, we did not perform external validation, and factors such as postmortem interval, skin pigmentation, and environmental effects could not be fully controlled. So, the results should be considered as preliminary and idea-generating, not as solid guidelines for forensic work. As more than one image depicting injuries may have been obtained from the same autopsy specimen, the conclusions drawn here pertain only to image-level observations.

Future studies should include larger and more balanced datasets with standardized forensic photography protocols. Automated image analysis techniques and external validation studies should be explored to improve reproducibility and generalizability. Additional research using advanced computational approaches may further strengthen the role of quantitative image analysis in forensic medicine.

## Conclusions

This exploratory study shows that ImageJ can be used for objective and repeatable morphometric and grayscale texture analysis in relation to postmortem injuries. Differences between the injury groups were statistically significant with respect to parameters such as area, perimeter, solidity, and gray-level SD. These differences indicate the potential use of these parameters as additional indicators. Still, all findings are merely exploratory and cannot be taken as definitive evidence. The use of ImageJ analysis should be seen as a complement to, but not a substitute for, visual analysis conducted by a forensic expert.
